# Infection by *Ralstonia* Species in Cystic Fibrosis Patients: Identification of *R. pickettii* and *R. mannitolilytica* by Polymerase Chain Reaction

**DOI:** 10.3201/eid0807.010472

**Published:** 2002-07

**Authors:** Tom Coenye, Peter Vandamme, John J. LiPuma

**Affiliations:** * University of Michigan Medical School, Ann Arbor, Michigan, USA; † Ghent University, Ghent, Belgium

**Keywords:** *Ralstonia pickettii*, *Ralstonia mannitolilytica*, cystic fibrosis, identification, molecular epidemiology, PCR, *Burkholderia cepacia* complex

## Abstract

The frequency of respiratory tract infections caused by *Ralstonia* species in persons with cystic fibrosis (CF) and the role of these species in CF pulmonary disease are not well documented. In part, this lack of documentation may be attributed to the difficulty in accurately identifying *Ralstonia* species; *R. mannitolilytica* and *R. pickettii* in particular may be misidentified as other closely related species, particularly those of the *Burkholderia cepacia* complex. We used polyphasic analyses to identify 42 *Ralstonia* isolates from sputum cultures from 38 CF patients. Several isolates that could not be identified to the species level may belong to novel *Ralstonia* species. We demonstrated chronic colonization by using genotyping of serial isolates recovered from the same patient. To facilitate identification of *R. mannitolilytica* and *R. pickettii*, we developed 16S ribosomal DNA-based polymerase chain reaction assays that allow sensitive and specific identification of these species.

Cystic fibrosis (CF) is the most frequent hereditary disease in Caucasian populations (1); chronic microbial colonization of the large airways, leading to exacerbations of pulmonary infection, is the major cause of illness and death in CF patients. Typical CF pathogens include *Staphylococcus aureus*, *Pseudomonas aeruginosa*, *Haemophilus influenzae,* and *Burkholderia cepacia* complex; other species, including *Stenotrophomonas maltophilia*, *Alcaligenes* (*Achromobacter*) *xylosoxidans*, *B. gladioli,* and *R. pickettii* have been recovered from sputum cultures of CF patients as well (2,3). Recently, we showed that a number of unusual bacterial species (including several novel species within the *α-Proteobacteria*) are also occasionally isolated from CF patients (4). Infection with mucoid *P. aeruginosa* and members of the *B. cepacia* complex is associated with increased illness and death in CF patients (5–7), but the clinical importance of infection with these other species is less clear.

The genus *Ralstonia* was proposed in 1995 (8). Since its creation, the taxonomy of the genus has expanded to include 11 species, which are *R. pickettii*, *R. solanacearum*, *R. eutropha*, *R. gilardii*, *R. paucula*, *R. basilensis*, *R. oxalatica*, *R. mannitolilytica*, *R. taiwanensis*, *R. campinensis*, and *R. metallidurans* (8–14). *Ralstonia* spp. are isolated from a wide variety of ecologic niches, including plants and soils contaminated with heavy metals. *R. pickettii* has been associated with nosocomial outbreaks caused by contaminated solutions used for patient care and with pseudoepidemics caused by contaminated solutions in the diagnostic laboratory (15–21). Several hospital-associated outbreaks attributed to *R. mannitolilytica* (formerly known as *R. pickettii* biovar 3 or *P. thomasii*) have been described (12,22,23). *R. paucula* and *R. gilardii* have only sporadically been isolated from human clinical samples, including cerebrospinal fluid, bone marrow, wounds, and the respiratory tract (9,10). A complete assessment of the frequency of human infection due to *Ralstonia* species is confounded by the difficulty in accurate species identification by using standard microbiologic techniques. Indeed, these species are frequently misidentified as *P. fluorescens* or *B. cepacia* complex (12,24–26).

We describe the occurrence of several *Ralstonia* species in the respiratory secretions of CF patients. We also describe the development and evaluation of two polymerase chain reaction (PCR) assays for rapid, accurate identification of *R. pickettii* and *R. mannitolilytica*.

## Materials and Methods

### Bacterial Strains and Study Population

Since early 1997, the *Burkholderia cepacia* Research Laboratory and Repository (University of Michigan, Ann Arbor, MI) has received more than 4,000 bacterial isolates, collected from CF patients receiving care in 145 CF treatment centers in 130 U. S. cities. Isolates received were tentatively identified by the referring microbiology laboratory as *B. cepacia* complex or a related species or were not identified to the species level. From these isolates, we identified 42 *Ralstonia* isolates obtained from 38 patients who had received care in 19 treatment centers in 18 U. S. cities. The type and reference strains of *Ralstonia*, *Pandoraea*, *Burkholderia*, *Alcaligenes*, and *Bordetella* species have been described (9–14). These strains were obtained from the BCCM/LMG-Bacteria Collection (Laboratorium voor Microbiologie, Universiteit Gent, Belgium) or were provided by D. Henry (University of British Columbia, Vancouver, Canada). All isolates were grown aerobically on Mueller-Hinton broth (Becton, Dickinson and Company, Cockeysville, MD) supplemented with 1.8% (wt/vol) agar and incubated at 32°C.

### Species Identification

We used a polyphasic approach to identify all isolates, including biochemical tests, 16S ribosomal (r)DNA-based PCR assays and sodium dodecyl sulfate-polyacrylamide gel electrophoresis (SDS-PAGE) of whole-cell proteins. Biochemical tests (determination of oxidase, lysine decarboxylase, and *o*-nitrophenyl-β-D-galactoside activity; growth on *B. cepacia* selective agar; and oxidation-fermentation of sucrose) were performed as described (27). SDS-PAGE of whole-cell proteins was performed as described (9,10), and isolates were identified by comparison with a database containing protein profiles of all *Ralstonia* species. We used 16S rDNA-based PCR assays (28) to determine whether or not isolates belonged to the genera *Burkholderia* or *Ralstonia* or to the *B. cepacia* complex.

### Genotyping of Serial Isolates

Multiple isolates from a single patient were genotyped by randomly amplified polymorphic DNA (RAPD) genotyping as described (29). We digitized gel images with a GelDoc2000 gel analyzer (Bio-Rad Laboratories, Hercules, CA) and stored them as tagged image files. After normalization with the molecular weight marker, patterns were analyzed with Molecular Analyst Fingerprinting Plus software (Bio-Rad Laboratories). Similarities between patterns were calculated by using Pearson’s product-moment correlation coefficient. We considered isolates to belong to the same genotype if they shared 90% or more similarity.

### Development of Primers for Species-Specific PCR Assays

We retrieved 16S rDNA sequences of all *Ralstonia* spp. and representatives of related genera from the GenBank database, using the MegAlign software package (DNASTAR Inc., Madison, WI) to align the sequences. Based on this alignment, we developed primers specific for *R. pickettii* and *R. mannitolilytica*: Rp-F1 (5´-ATGATCTAGCTTGCTAGATTGAT-3´) and Rp-R1 (5´-ACTGATCGTCGCCTTGGTG-3´) (forward and reverse primers for the identification of *R. pickettii*) and Rm-F1 (5´-GGGAAAGCTTGCTTTCCTGCC-3´) and Rm-R1 (5´-TCCGGGTATTAACCAGAGCCAT-3´) (forward and reverse primers for the identification of *R. mannitolilytica*).

### Polymerase Chain Reaction

DNA was prepared as described (30). PCR assays were performed in 25-µL reaction mixtures, containing 2 µL DNA solution, 1U *Taq* polymerase (GIBCO Invitrogen Corp., Gaithersburg, MD), 250 mM (each) deoxynucleotide triphosphate (GIBCO Invitrogen Corp.), 1.5 mM MgCl_2_, 1x PCR buffer (GIBCO Invitrogen Corp.), and 20 pmol of each oligonucleotide primer. Amplification was carried out with a PTC-100 programmable thermal cycler (Labtrade Inc., Miami, FL). After initial denaturation for 2 min at 94°C, 30 amplification cycles were completed, each consisting of 1 min at 94°C, 1 min at 55°C (for identifying *R. pickettii*) or 57°C (for identifying *R. mannitolilytica*), and 1 min 30 s at 72°C. A final extension of 10 min at 72°C was applied. Negative control PCRs with all reaction mixture components except template DNA were used for every experiment.

### Evaluation of the PCR Assays

For evaluating PCR assays, we tested 152 isolates, including 79 *Ralstonia* isolates (both clinical isolates and reference strains) and 73 isolates representing phylogenetically related species that may be found in sputum cultures of CF patients. Isolates tested were, as follows: *R. pickettii* (27 isolates), *R. mannitolilytica* (34), *R. gilardii* (4), *R. paucula* (2), *R. taiwanensis* (1), *R. basilensis* (1), *R. eutropha* (1), *R. oxalatica* (1), *R. solanacearum* (1), *R. campinensis* (1), *R. metallidurans* (1), *Ralstonia* sp. (5), *B. cepacia* genomovar I (3), *B. multivorans* (2), *B. cepacia* genomovar III (7), *B. stabilis* (2), *B. vietnamiensis* (2), *B. cepacia* genomovar VI (5), *B. ambifaria* (3), *B. gladioli* (6), *B. fungorum* (1), *Pandoraea apista* (5), *P. norimbergensis* (3), *P. pnomenusa* (2), *P. sputorum* (4), *P. pulmonicola* (2), *Alcaligenes xylosoxidans* (5), *P. aeruginosa* (5), *S. maltophilia* (5), and one isolate each of *A. denitrificans*, *A. piechaudii*, *A. faecalis*, *A. ruhlandii*, *Bordetella avium*, *B. hinzii, B. trematum, B. bronchiseptica*, *B. pertussis*, *B. parapertussis,* and *B. holmesii*.

## Results

### Species Identification

Isolates were tentatively identified as belonging to the genus *Ralstonia* if they 1) reacted with primer pair RHG-F/RHG-R (specific for *Burkholderia* and *Ralstonia* spp.) (28), 2) showed no lysine decarboxylase and *o*-nitrophenyl-β-D-galactoside activity, 3) produced no acid from sucrose, and 4) showed oxidase activity. Using these criteria, we identified 42 putative *Ralstonia* sp. isolates. These isolates were further identified to the species level by using SDS-PAGE of whole-cell proteins. Most isolates (25) were identified as *R. mannitolilytica*; 9 were identified as *R. pickettii*. Two isolates were identified as *R. gilardii,* and another as *R. taiwanensis*. Five isolates clearly belonged to the genus *Ralstonia* but could not be classified into one of the known species. Pending further investigations, these isolates were classified as *Ralstonia* sp.

### Genotyping of Serial Isolates

**Figure 1:**
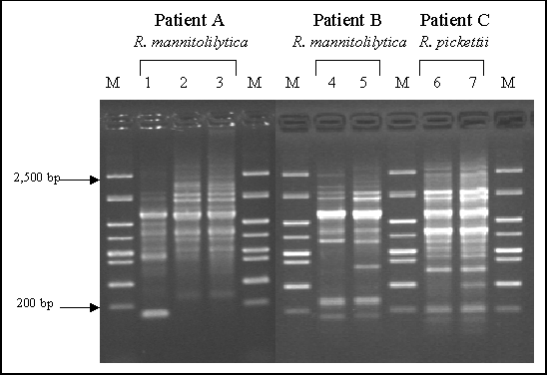
Randomly amplified polymorphic DNA analysis of serial isolates from three patients. M: molecular weight marker; lanes 1, 2, and 3: serial *Ralstonia mannitolilytica* isolates from patient A in chronological order; lanes 4 and 5: serial *R. mannitolilytica* isolates from patient B in chronological order; and lanes 6 and 7: serial *R. pickettii* isolates from patient C in chronological order.

We identified two patients (A and B) who were sputum-culture positive for *R. mannitolilytica* and one patient (C) who was culture positive for *R. pickettii* on more than one occasion. The three *R. mannitolilytica* isolates cultured from patient A were recovered over a period of >2 years. RAPD genotyping indicated that the first isolate clearly differed from the two isolates recovered subsequently; the latter two isolates (recovered 20 months apart) were the same genotype (Figure 1). Similarly, the two *R. mannitolilytica* isolates recovered from patient B (cultured 8 weeks apart) were the same genotype, as were the two *R. pickettii* isolates recovered from patient C (cultured 6 weeks apart) (Figure 1).

### Primer Design

**Figure 2:**
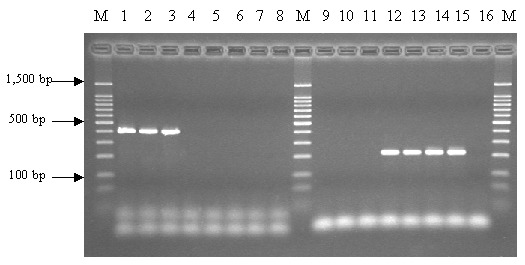
Polymerase chain reaction analysis of *Ralstonia* strains with primer pairs Rm-F1/Rm-R1 (lanes 1–8) and Rp-F1/Rp-R1 (lanes 9–16). M: 100-bp DNA ladder; lanes 1, 2, 3, 9, 10, and 11: *R. mannitolilytica*; lanes 4, 5, 6, 7, 12, 13, 14, and 15: *R. pickettii*; and lanes 8 and 16: *R. gilardii*.

Alignment of 16S rRNA gene sequences of *Ralstonia* sp. available in GenBank showed similarity values >93.1% and >98.2% within the species *R. pickettii* and *R. mannitolilytica*, respectively. Identity of sequences between these two species ranged from 89.9%–96.8%. Several species-level sequence signatures were detected and were incorporated into the species-specific primers Rp-F1 and Rp-R1 (forward and reverse primer for *R. pickettii*) and Rm-F1 and Rm-R1 (forward and reverse primer for *R. mannitolilytica*). PCR with these primers resulted in the amplification of fragments of 210 bp and 398 bp, respectively (Figure 2). Each of the 152 strains included in this study was examined by PCR with the primer pairs described (Table).

**Table:** Sensitivity and specificity of polymerase chain reaction (PCR) assays for the identification of *Ralstonia mannitolilytica* and *R. pickettii*

			No. of strains	
Primer pair and species tested	Sensitivity (%)	Specificity (%)	Positive	Negative
Rp-F1/Rp-R1				
R. pickettii *(n=27)*	89	99	24	3^a^
All others (n=125)			1^b^	124
Rm-F1/Rm-R1				
*R. mannitolilytica* (n=34)	100	99	34	0
All others (n=118)			1^c^	117

^a^Of the three *R. pickettii* isolates that gave a false-negative reaction in this PCR test, two were identified as *R. pickettii* by the referring laboratory, while one was received unidentified. ^b^One unidentified *Ralstonia* sp. isolate cross-reacted with this primer pair. ^c^One *R. pickettii* isolate cross-reacted with this primer pair.

## Discussion

The occurrence and clinical role of *Ralstonia* sp. in the respiratory secretions of persons with CF have not been systematically investigated because of the rapidly changing taxonomy of the genus *Ralstonia* and the absence of rapid, reliable methods for species identification. We used a polyphasic approach to identify *Ralstonia* sp. in sputum cultures of CF patients and developed two PCR assays for identifying *R. pickettii* and *R. mannitolilytica*.

Previous reports describing the bacterial flora of the respiratory tract of CF patients have focused mainly on *P. aeruginosa* and *B. cepacia* complex organisms (3,5,31); reports describing the presence of *Ralstonia* species in sputum cultures of CF patients are scarce and often anecdotal. In a prospective study, Burns et al. (2) isolated *R. pickettii* from only 2 of 559 patients. More recently, we have shown that other *Ralstonia* species, including *R. mannitolilytica*, *R. taiwanensis*, and *R. gilardii,* can also be isolated from the respiratory secretions of CF patients (4). In this study, we identified *Ralstonia* species recovered from sputum cultures of 38 CF patients. Collectively, these data indicate that the prevalence of *Ralstonia* sp. in the CF population is rather low. However, because we did not specifically survey all referring laboratories for all *Ralstonia* species that may have been recovered from CF specimens, we were not able to define a more precise prevalence of *Ralstonia* sp. in the CF population.

Our data do not provide evidence for patient-to-patient spread of *Ralstonia* sp. because no clustering of cases occurred within centers or geographic regions (data not shown). However, we were able to document persistent colonization with *Ralstonia* species in three patients. Patient A’s infection is particularly interesting. In this patient, an initial *R. mannitolilytica* strain was apparently replaced with another strain, which then persisted for >20 months. However, the bacterial and host factors involved in infection by more that one *R. mannitolilytica* strain or with chronic colonization remain to be defined.

Five *Ralstonia* isolates could not be identified to the species level. 16S rDNA PCR and SDS-PAGE of whole-cell proteins clearly indicated that these isolates belong to the genus *Ralstonia*, suggesting that they may represent novel *Ralstonia* sp. Further polyphasic taxonomic studies are needed to clarify their status. The finding of *R. mannitolilytica*, *R. gilardii*, *R. taiwanensis,* and possible novel *Ralstonia* species in respiratory secretions of CF patients suggests that these organisms may be emerging human pathogens and again highlights the fact that the bacterial biodiversity in the respiratory tract of CF patients has thus far been underestimated (4).

Of the 25 *R. mannitolilytica* strains identified in this study, 9 were initially identified by the referring laboratory as *R. pickettii*, 8 as *B. cepacia* complex, 6 as *Burkholderia* sp., 1 as *B. gladioli*, and 1 as *P. fluorescens*. Of the 9 *R. pickettii* strains identified, 3 were identified by the referring laboratory as *R. pickettii*, 2 as *Burkholderia* sp., 1 as *Pseudomonas* sp., 1 as *B. cepacia* complex, and 2 isolates as unidentified. The *R. gilardii* and *R. taiwanensis* isolates were received as *B. cepacia* complex and *S. maltophilia*, respectively. Most (81%) of these isolates were capable of growth on *B. cepacia* selective agar. These observations reiterate that identification of these species is not straightforward and that their misidentification as other CF pathogens, such as *B. cepacia* complex, is not uncommon. Such misidentification has an important impact on infection control in CF since the efficiency of these measures depends on accurate identification of the microorganisms involved. Infection-control policies, particularly those recommended to prevent interpatient spread of *B. cepacia* complex, have a tremendous impact on the quality of life of CF patients (6,7). To enhance accurate identification of CF pathogens, several PCR assays have been developed recently (28,30,32–35). We sought to design similar PCR tests to allow the identification of *R. pickettii* and *R. mannitolilytica* based on species-level signature sequences in the 16S rRNA gene. By comparing available *R. pickettii* and *R. mannitolilytica* 16S rRNA gene sequences with sequences from other *Ralstonia* species and representatives of the phylogenetically closely related genera *Burkholderia* and *Pandoraea*, we identified several regions that showed sufficient diversity to allow the design of primer pairs Rp-F1/Rp-R1 and Rm-F1/Rm-R1, permitting the sensitive and specific identification of *R. pickettii* and *R. mannitolilytica*, respectively (Table).

The results of our study indicate that a number of *Ralstonia* species can be isolated from sputum cultures of CF patients. The correct identification of these species presents a challenge for diagnostic microbiology laboratories. Our study supports the use of genotypic methods to augment routine phenotypic evaluation. The combined use of the two PCR assays described will allow the identification of most *Ralstonia* species encountered in sputum cultures of CF patients. Most importantly, the use of these assays will substantially reduce the misidentification of *R. pickettii* and *R. mannitolilytica* as *B. cepacia* complex. These tests will be a valuable adjunct in the evaluation of CF sputum culture isolates and will allow more precise study of the prevalence and natural history of human infection by these emerging pathogens.
